# Heterotopic ossification of the esophagus identified during surgical management of esophageal carcinoma

**DOI:** 10.1093/jscr/rjag538

**Published:** 2026-07-31

**Authors:** Grace Gregory, Justin Watson, Joseph Krenzer

**Affiliations:** University of Washington School of Medicine, 1410 NE Campus Parkway, Seattle, WA 98195, United States; Billings Clinic, 2800 10th Ave N, Billings, MT 59101, United States; Billings Clinic, 2800 10th Ave N, Billings, MT 59101, United States

**Keywords:** heterotopic ossification, esophagus, esophageal carcinoma, Barrett’s esophagus, chronic inflammation

## Abstract

Heterotopic ossification (HO) is the formation of mature lamellar bone within soft tissues and is most commonly associated with trauma or neurologic injury. Gastrointestinal involvement is exceedingly rare, with only one prior reported case of esophageal HO in association with Barrett’s esophagus. The pathogenesis remains poorly understood but is thought to involve chronic inflammation and dysregulated mesenchymal cell differentiation. We report a rare case of heterotopic ossification of the esophagus identified during the diagnostic evaluation and surgical management of esophageal squamous cell carcinoma following neoadjuvant chemoradiation. Intraoperatively, a firm, foreign body–like structure was encountered at the tumor site and was histologically confirmed as mature heterotopic bone without residual carcinoma. This represents the second reported case of esophageal HO and the first described in association with malignancy. Recognition of this entity is important, as heterotopic bone may complicate surgical dissection and mimic malignant invasion, with potential implications for staging and operative planning.

## Introduction

Heterotopic ossification (HO) is characterized by the formation of mature lamellar bone in soft tissues where bone is not normally present. Risk factors include neurologic injury, musculoskeletal trauma, thermal injuries, and surgical procedures. While specific mechanisms in HO remain unclear, proposed mechanisms include persistent inflammation after trauma and dysregulated bone morphogenetic protein signaling of mesenchymal progenitor cells into osteoblasts [1].

HO within the gastrointestinal tract is rare. To date, the Haque *et al*. case series remains the only identified report documenting esophageal HO in association with Barrett’s esophagus without associated neoplasia [2]. In this case report, we describe a rare case of esophageal HO identified during the evaluation and surgical management of esophageal carcinoma. This represents only the second reported instance of esophageal HO and the first described in association with malignancy.

## Case report

A 72-year-old male with a history of hypertension, hyperlipidemia, polycystic kidney disease, gastroesophageal reflux disease, and alcohol and tobacco use disorders presented with minimal dysphagia and an unintentional 50-pound weight loss over several months.

An initial positron emission tomography/computed tomography (PET/CT) performed for pulmonary nodule evaluation revealed non-hypermetabolic nodules but identified hypermetabolic activity within a mid-esophageal mass, alongside mediastinal, porta hepatis, and inguinal lymphadenopathy (Fig. 1). Upper endoscopy demonstrated a partially obstructing, circumferential, fungating mass at 30–35 cm from the incisors, exhibiting active and recent stigmata of bleeding. Cold forceps biopsies were obtained. Histopathological analysis revealed a high-grade spindle and epithelioid malignant neoplasm, cytokeratin-positive, most consistent with invasive keratinizing squamous cell carcinoma (Fig. 2).

**Figure 1:**
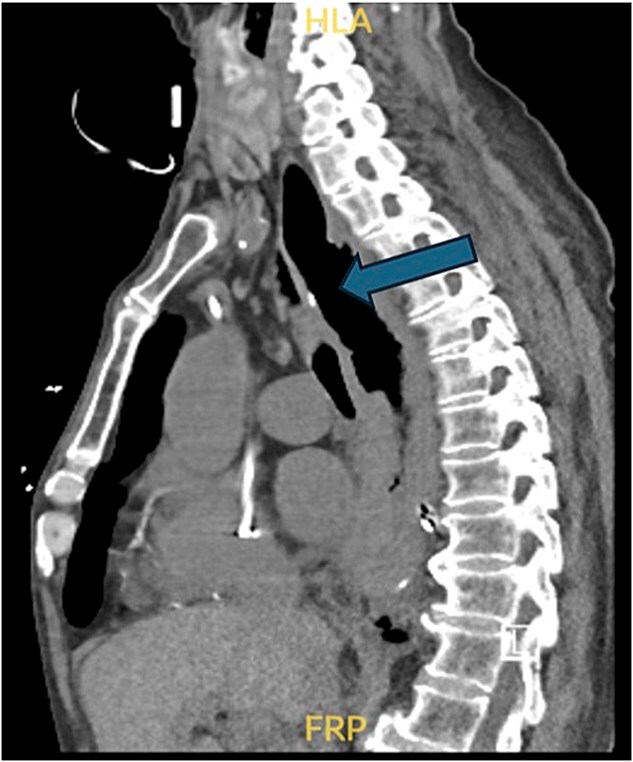
Sagittal CT demonstrating focal intramural calcification within the mid-esophageal mass (arrow), later confirmed to represent heterotopic ossification.

**Figure 2:**
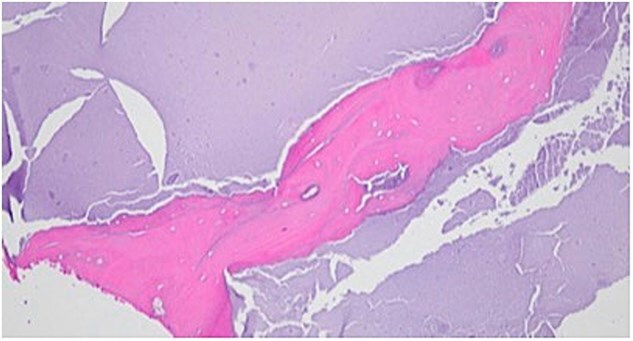
Histopathologic section demonstrating heterotopic ossification with mature lamellar bone formation (10× magnification).

The patient underwent neoadjuvant chemoradiation with 223.8 mg carboplatin and 88.8 mg paclitaxel administered over 5 cycles, along with 28 fractions of intensity-modulated radiation therapy (IMRT) to a total dose of 50.6 Gy, followed by minimally invasive esophagogastrostomy after restaging PET imaging. Intraoperatively, a dense adhesion between the esophagus and adjacent intrathoracic structures, particularly the posterior membranous left mainstem bronchus, was encountered at the known tumor site (Fig. 3). During dissection, a firm, foreign body–like structure was encountered and excised; histopathologic evaluation confirmed mature heterotopic bone without evidence of residual carcinoma (Fig. 2). Final pathology demonstrated a T0N1 stage with significant treatment response, with residual tumor identified in 2 of 12 resected lymph nodes.

**Figure 3:**
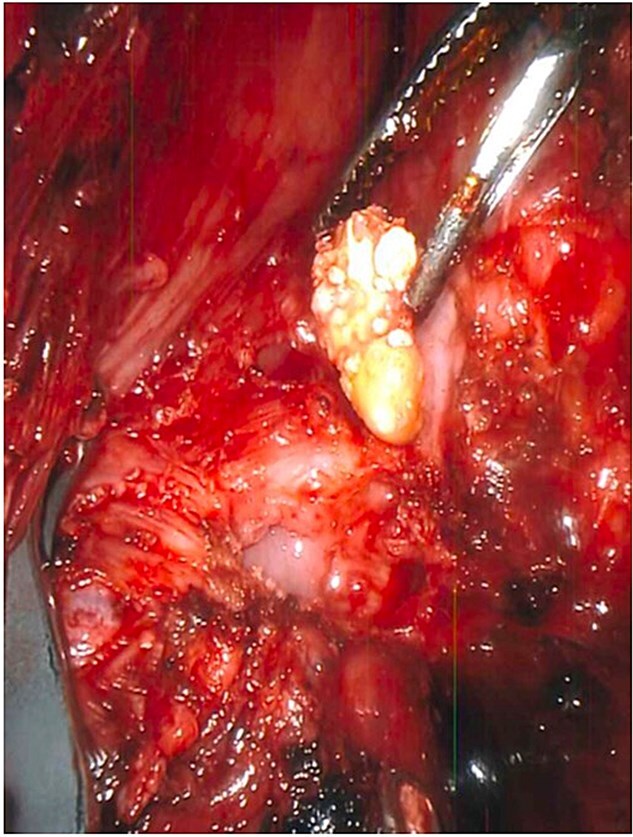
Intraoperative identification of heterotopic ossification at the mid-esophageal tumor.

## Discussion

This case highlights the rare phenomenon of HO within the esophagus, discovered incidentally during the diagnostic workup and surgical management of esophageal carcinoma. To date, only one prior case of esophageal HO has been reported in the literature. In 1996, Haque and Eisen described a patient with Barrett’s esophagus who presented with nausea and vomiting. Endoscopy with biopsy of an esophageal ulcer revealed immature bone formation at the ulcer base. The authors postulated that HO developed as a consequence of chronic inflammation, independent of any direct stimulatory influence from neoplastic epithelial cells [2].

The pathogenesis of bone formation within the gastrointestinal tract remains poorly understood. Proposed mechanisms include metaplastic transformation of epithelial or mesenchymal cells, tumor-associated stromal changes, and treatment-related effects, particularly radiotherapy, that activate osteogenic signaling pathways [3]. Heterotopic ossification in the gastrointestinal tract most commonly occurs in the colorectum, particularly in association with mucinous adenocarcinomas, making esophageal involvement particularly rare [2]. In the setting of esophageal malignancy, the convergence of chronic inflammation, tissue injury, and neoplastic processes provides a plausible biological environment for the development of heterotopic ossification.

Clinically, the presence of HO within the esophagus has important implications. While histologic identification of osseous tissue is typically straightforward, correlation with intraoperative and radiographic findings is critical, particularly when tumor closely approximates or involves heterotopic bone, to avoid misinterpretation as tumor invasion into bone and subsequent overstaging of the pathologic tumor stage. Heterotopic bone may also complicate surgical dissection and mimic malignant osseous invasion on imaging or histopathologic evaluation [2]. This is especially relevant in patients undergoing surgical resection for esophageal squamous cell carcinoma who have received prior radiotherapy, where post-treatment changes further confound pathologic interpretation.

Radiation therapy has a complex, dose-dependent relationship with heterotopic ossification. Low-dose radiation (~7 Gy), commonly administered perioperatively in high-risk orthopedic procedures, is effective in preventing HO formation by suppressing osteogenic signaling pathways. In contrast, higher cumulative doses used in oncologic treatment have been associated with late radiation-induced calcification and ossification, typically occurring years after exposure [4]. The patient in this case received 50.6 Gy of IMRT, a dose within the range reported to produce delayed heterotopic calcification. Although the temporal relationship and causality cannot be definitively established, prior radiotherapy may have contributed to the development of heterotopic bone in this setting [5].

Management of esophageal HO is generally guided by the underlying pathology rather than the heterotopic bone itself. There are currently no established guidelines specific to HO in the esophagus [2]. Surgical excision may be considered if the lesion is symptomatic or interferes with oncologic resection. The literature underscores the importance of distinguishing heterotopic ossification from true tumor invasion into bone, as this distinction carries significant implications for staging, prognosis, and treatment planning [2]. Although rare, increased awareness of gastrointestinal HO is essential for accurate diagnosis and intraoperative decision-making. Further research is needed to clarify the mechanisms, clinical significance, and optimal management of this unusual entity.
